# Extension for Community Healthcare Outcomes (ECHO) Telementoring in the Military: Where We Are Now, Opportunities and Challenges

**DOI:** 10.1093/milmed/usab010

**Published:** 2021-08-28

**Authors:** Joanna Katzman, Laura Tomedi, Robin Swift, Erick Castillo, Connie Morrow, Laurie Lutz, Kevin T Galloway, Kimberly McCoy-Stafford, Zachary Klein, Greg Turner, Darrick J Beckman, Jennifer Terrell, Shannon Forde, Chamron Martin, Sharon Morgan

**Affiliations:** ECHO Institute, University of New Mexico, Albuquerque, NM 87102, USA; ECHO Institute, University of New Mexico, Albuquerque, NM 87102, USA; ECHO Institute, University of New Mexico, Albuquerque, NM 87102, USA; Naval Medical Center San Diego, San Diego, CA 92134, USA; Air Force Diabetes Center of Excellence, JBSA-Lackland, AFB, TX 78236, USA; Defense Health Headquarters, Falls Church, VA 22040, USA; Strategic Communications and Policy, Defense & Veterans Center for Integrative Pain Management, Rockville, MD 20852, USA; Defense Health Agency, Falls Church, VA 22041, USA; ASRC Federal, Reston, VA 20190, USA; ASRC Federal, Reston, VA 20190, USA; Air Force Diabetes Center of Excellence, JBSA-Lackland, AFB, TX 78236, USA; Naval Medical Center San Diego, San Diego, CA 92134, USA; Naval Medical Center San Diego, San Diego, CA 92134, USA; ECHO Institute, University of New Mexico, Albuquerque, NM 87102, USA; Connected Health Branch Clinical Support Division, Medical Affairs, Defense Health Agency, Falls Church, VA 22041, USA

## Abstract

**Introduction:**

In collaboration with the ECHO (Extension for Community Healthcare Outcomes) Institute since 2012, the Army, Navy, and Air Force have developed medical teleECHO programs to address various health and safety issues affecting military personnel. This article describes and compares the current state of military teleECHOs as well as the growth and change over time.

**Materials and Methods:**

This study evaluated continuing education units (CEUs) offered, average session attendance, and number of spoke sites for current military teleECHO programs across the service branches.

**Results:**

Between 2012 and 2019, the military teleECHO initiative grew from one program to seven different teleECHO programs, covering topics from pain to diabetes to amputee care. Military ECHOs now provide training to 10 countries and 27 states in the United States. Between October 2018 and September 2019, the military ECHO programs provided a total of 51,769 continuing medical education (CME) hours to a total of 3,575 attendees from 223 spoke sites.

**Conclusions:**

The military has successfully used the ECHO model to improve the health and safety of active-duty military, retirees, and dependents.

## INTRODUCTION

### Brief Overview of ECHO

Project ECHO (Extension for Community Healthcare Outcomes) began in 2003 at the University of New Mexico (UNM) to leverage technology and improve specialty care access in New Mexico’s rural and medically underserved communities. The ECHO model features an interprofessional team of specialists at the “hub” site, training primary care clinicians at the “spoke” sites remotely using videoconferencing technology. The spoke clinicians present real, de-identified cases in an “all teach, all learn” format, while the participants are encouraged to engage in the bi-directional best practices learning environment. Short lectures and no-cost continuing medical education (CME) credits are provided in all teleECHO sessions and outcomes are evaluated.^1^ Currently, there are 401 ECHO hubs worldwide, in 49 states and 40 countries. The ECHO model is now addressing over 70 health-related and non-medical conditions.^2^

The opioid epidemic is a serious public health issue that has cost the USA $1.02 trillion.^3^ Project ECHO’s Chronic Pain and Opioid Management program (ECHO Pain) is staffed by an interprofessional team of subject matter experts including neurology, myofascial pain, addiction psychiatry, behavioral health, physical therapy, chiropractic medicine, and advanced practice clinicians. Founded in 2009, ECHO Pain teaches non-opioid pain management, safe opioid prescribing, and screening for opioid use disorder and consults on pain and opioid misuse cases in weekly 90-min sessions.

### Military ECHOs

Beginning in 2012, the U.S. Army collaborated with the ECHO Pain team to launch a large-scale replication of ECHO Pain across five U.S. Army Medical Command (MEDCOM) regions. The Army Comprehensive Pain Management Program^4^ was invested in maximizing the role of primary care providers in managing chronic pain. Three Army Pain ECHO hubs were created in the continental USA, one in Hawaii, and one in Germany. The Hub Readiness Replication Model^4^ was created during this process and was the first example of a widely used replication tool. The goals of this tool were to ensure: (1) fidelity to the ECHO model during each MEDCOM teleECHO Pain launch, (2) consistency in the replication process, and (3) adaptability for use during all future clinical and non-clinical replications.

Using the same Hub Readiness Replication Model^4^ as the U.S. Army, the U.S. Navy launched two large Navy Pain ECHOs (Navy Medicine East in Portsmouth, Virginia, and Navy Medicine West in San Diego, California) as part of their comprehensive pain program.^5^ The Navy Polypharmacy ECHO started as an onboarding effort for 21 new pain-focused clinical pharmacists serving primary care medical home clinics. This program focuses on treating complex pharmacy pain patients and has grown to become a virtual network for the Navy and other military branches both in the continental USA and overseas. The Navy also developed a Traumatic Brain Injury (TBI) ECHO to expand and increase resources associated with TBI medical knowledge across the military healthcare system. The TBI ECHO program was initially created to support the Naval Primary Care Clinicians located within the operational commands to better enable them to treat sailors and marines who suffer TBI-related injuries. Finally, the Navy most recently launched the Navy Sexual Assault Medical Forensics Examination ECHO to standardize the documentation processes and requirements associated with conducting sexual assault medical forensic examinations across Navy Medicine.

Air Force Diabetes ECHO was launched at the Diabetes Center of Excellence at Wilford Hall Ambulatory Surgical Center and Lackland Air Force Base to address limited endocrinology access at small military treatment facilities (MTFs).^6^ This telementoring program provides the ability to reach deployed personnel, supporting force readiness regarding diabetes prevention and pre-diabetes.

The Amputation Care ECHO was originally launched as an Extremity Trauma and Amputation Center of Excellence initiative designed to address educational gaps and facilitate a knowledge sharing network across the Military Health System with clinicians involved in amputation care. Initially, Amputation Care ECHO was established with one hub, Walter Reed National Military Medical Center, and four spokes, including the Veterans Health Administration (VHA).

Please see Table I for detailed information related to Army, Navy, Air Force, and Amputation Care ECHO programs (date launched, hub team composition, location, target audience, session details, number of spokes and attendees, and number of CMEs available).

**TABLE I.** Descriptions of Army, Navy, Air Force, and Amputation Care Extension for Community Healthcare Outcomes (ECHO) Programs, October 2018-September 2019

	Amputation care	Air force	Navy	Army
Date launched	October 2017	January 2012	Pain: 2014-2015	2012-2014
			TBI: November 2017	
			PPI: October 2015	
			SAMFE: January 2019	
Expertise on hub team	Physiatrists, behavioral health specialists, physical and occupational therapists, orthotists, prosthetists, and other experts involved in amputation care as needed.	Endocrinologist, registered nurse, and an education specialist	Pain: Pain specialist, pharmacist, physical therapist, social workers, and nurses TBI: Neurologists, social worker, neuropsychologists, and physical and occupational therapist PPI: Pharmacy department leads, clinic managers, and clinical pharmacists SAMFE: Regional SAMFE leaders and program managers	Pain specialist, clinical psychologist, social workers, nurse, pharmacist, physical therapists and occupational therapists and technicians, and massage therapist
Hub location	Walter Reed Medical Center	Wilford Hall Ambulatory Surgical Center and Lackland Air Force Base	Pain: Navy Medicine East in Portsmouth, VA, and Navy Medicine West in San Diego, CA	Brooke, Dwight D. Eisenhower, Madigan, Tripler, Womack, and Landstuhl Medical Centers
			TBI: Navy Medicine West, Camp Pendleton	
			PPI: Navy Medicine East	
			SAMFE: Navy Medicine East	
Target audience	Physical therapists, occupational therapists, orthotists, prosthetists, and physiatrists	Primary care clinicians and nurses, technicians, pharmacists, dietitians, and nursing disease managers	Pain: Primary care clinicians TBI: PCPs and case managers PPI: Pharmacists and pharmacy technicians SAMFE: Primary care clinicians and forensic medical examiners	Primary care clinicians, nurse case managers, physical and rehabilitation technicians, and behavioral health consultants
Length of session	60 min	60 min	Pain: 60 min	90 min
			TBI: 60 min	
			PPI: 90 min	
			SAMFE: 90 min	
Frequency of each session	Once a month	Twice a month	Pain: once a week	Once a week
			TBI: once a month	
			PPI: once a month	
			SAMFE: once a month	
Number of spokes	11	48	92	72
Number of attendees	451	607	1,896	621
Number of CMEs available	9,471	607	1,650	40,041

Abbreviation: CME, continuing medical education; TBI, traumatic brain injury; PCP, primary care provider; SAMFE, sexual assault medical forensics examination.

### Fidelity to the ECHO Model

During the U.S. Army–Project ECHO collaboration, MOCK ECHO and Anatomy of an ECHO were both created as important tools to ensure fidelity and standardization across the six large Army Pain ECHO hubs, as well as to aid in the training of hub and spoke clinicians.^4,7^ The Anatomy of an ECHO training tool has been used as a guide to illustrate the process of presenting a case, asking for clarifying questions; developing a differential diagnosis; and making non-pharmacological, pharmacological, and interventional recommendations.^7^ MOCK ECHO is a simulation training that allows clinicians to practice presenting cases with an interprofessional hub team and multiple spoke clinicians. It assists with new military ECHO launches as well as with refresher trainings.^7^

### Curriculum Development

Because ECHO programs rely on evidence-based and evidence-guided treatment guidelines and recommendations, the development of a core ECHO curriculum is essential to training primary care clinicians in best practices to ensure the highest quality of care for their patients. One example of an ECHO curriculum that is being used by the Army and Navy Pain ECHOs is the Joint Pain Education Project (JPEP)—A collaboration between the Department of Defense (DoD) and VHA’s joint strategy on addressing the unwarranted variability of pain management practice. The JPEP common core curriculum consisted of 18 modules targeting the most common and challenging pain care conditions treated in primary care (Table II). Developed by subject matter experts within the DoD and VHA, the length and outline of each JPEP module were formatted so that it could be easily integrated into all appropriate education and training programs, specifically the VHA-ECHO and DoD ECHO initiatives for pain management. The JPEP modules were similarly structured to include the following key sections for each pain condition: anatomy and physiology, assessment and documentation, red flags, and recommendations on how to treat and when to refer.

**TABLE II.** The 18 Modules of the Joint Pain Education Project (JPEP) Common Core Curriculum

Understanding Pain Video
Modern Understanding of Pain
Pain Taxonomy and Physiology
DoD/VHA Stepped Care Model for Pain Care Recovery
Assessment of Pain
Assessment Tools
Acetaminophen, NSAIDs, and Opioids
Adjuvant Medications
Chronic Opioid Therapy Risk Evaluation and Mitigation
Behavioral Management of Chronic Pain
Provider Communication in Chronic Pain
Physical-Based Therapeutic Approaches to Pain Management
Integrative Pan Medicine
Pain Medicine Specialty Care
Neck Pain
Acute Low Back Pain
Chronic Low Back Pain
Shoulder Pain
Hip Pain
Knee Pain
Myofascial, Connective Tissue, and Fibromyalgia Pain
Central Neuropathic Pain
Peripheral Neuropathic Pain
Headache Pain
Visceral Pain
Psychiatric Comorbidities and Pain
Geriatric Pain
Palliative and Oncologic Pain Care
Women Pain Related Issues
Opioids and Pregnancy
Female Pelvic Pain

### Military ECHO Refresher Courses

Military ECHO refresher courses are offered to each of the current Air Force, Army, and Navy ECHOs by the faculty of the ECHO Pain team in order to provide ongoing continuing education related to the ECHO model. These refresher courses are provided both in-person and through synchronous and asynchronous videoconferencing. The content is not limited to, but may include MOCK ECHO simulation trainings, how to deliver Anatomy of an ECHO, helping hub teams strategize options for recruitment of spoke clinicians, and videoconferencing etiquette. Refresher courses allow the military ECHOs to preserve fidelity, maintain consistency, and assure standardization as clinicians and support teams rotate on and off of service.

## RESULTS

Since the DoD ECHO programs began in 2012, there are now hubs and spokes located in 27 U.S. states and 10 countries. In addition, many DoD ECHO programs collaborate across federal agencies to hold joint ECHO programs with the VHA and civilian academic medical centers. Some military ECHO clinics are well established and have been running for 8 years, whereas others are newer and have only been running for a year (Table I). Hub teams include specialists and experts to meet the individual needs of the clinics. Sessions are generally short, 60-90 min, to more easily fit into clinicians’ schedules. Frequency of sessions depends on needs of participants and availability of the hub team and ranges from once a month to once a week. Spoke sites vary in their attendance. Some ECHO programs have many attendees per spoke and this usually suggests that the participants meet together in a conference room. Other ECHO programs have participants alone or with one other clinician.

The number of attendees and spokes for the Navy reflects all Navy programs, including Navy Pain, Navy TBI, Navy PPI, and Navy SAFME. During the fiscal year 2019, the military ECHO programs provided a total of 51,769 CME hours to attendees. These are reflective of CMEs made available to, but not necessarily collected by, attendees. Each military service has a point of contact in their training and education section, who reviews and approves CME. The Army Pain ECHOs, which have been running the longest, made 40,041 CMEs available to attendees.

### Military/Veterans Affairs ECHO Research

The continued success of the original Army and Navy Pain ECHO programs are most likely due to deliberate programmatic pre-planning and initial trainings used in the Hub Readiness Replication Model as well as continued refresher courses. In a 4-year observational cohort study, the patients of clinicians who participated in Army and Navy Pain ECHO programs had greater declines in opioid-related prescriptions than patients whose providers opted not to participate.^8^ Using central Defense Health Agency (DHA) pharmacy data, new annual opioid prescriptions for patients whose clinicians participated in ECHO Pain versus the comparison group had a 23% (vs. 9%) reduction, *P* < .001.^8^ In addition, other military Pain ECHO programs that were originally trained using the Hub Readiness Model have been evaluated. Flynn et al. demonstrated that patients whose primary care providers participated in the telementoring program also had lower doses of prescribed opioids.^9^

The Air Force Diabetes ECHO program found that, after training, the average self-score for (1) perceived level of knowledge and (2) personal confidence level with regard to treating complex diabetes patients increased for all respondents.^6^ In this same study, 96.8% of primary care physicians reported that they were likely to make changes to their treatment practice for patients with diabetes based on what they learned in the ECHO program.

Many other federal agencies have adopted the ECHO model including the Veterans Affairs (VA) and the Indian Health Service (IHS). The VA has developed more than 10 Specialty Care Access Network (SCAN) ECHO programs, beginning in 2011. A recent study at the Ann Arbor, Michigan, VA has shown improved survival of patients with liver disease if clinicians use SCAN-ECHO as compared to usual care.^10^ For VA patients with chronic pain and opioid substance use disorder, SCAN-ECHO has proven beneficial in lowering opioid dose while incorporating a strong biopsychosocial model of care.^11^ In another large VA SCAN-ECHO pain study, the authors found that clinicians who present their patients to SCAN-ECHO for pain had an increase in referrals to rehabilitation and for non-opioid medications suggesting safer approaches to pain care. The study also concluded that primary care clinicians learned best practices pain management while presenting cases to SCAN-ECHO.^12^ An IHS ECHO telementoring study has shown significant benefit in clinician knowledge, confidence, and attitudes toward treating patients with chronic pain and addiction after training more than 1,300 clinicians.^13^

### Challenges and Opportunities

The ECHO model has proven to be a useful educational tool to support standardization of training and mentorship, as demonstrated by mock ECHO sessions conducted by organizations replicating the ECHO model.^7^ Tools have been developed specifically for military personnel to support fidelity of the program.^4^ Studies comparing the cost of pain management for patients using the ECHO model vs. in clinic found that, although the costs per patient were similar ($332.89 in clinic vs. $376.48 telehealth), following the ECHO model provided faster access to interdisciplinary and collaborative consultation while minimizing burden on patients.^14^ The ECHO telementoring in the military can leverage scarce specialty care resources to rapidly share emerging best practices and guidelines. Earlier recognition, treatment, and prevention of chronic pain, substance use, diabetes, TBI, sexual assault sequelae, and postamputation complications can each improve force readiness, which is a priority for all military commands. Additionally, military ECHOs provide training on subjects that are used as performance measures for healthcare facilities. For example, the Air Force, which runs the Diabetes ECHO, had A1c testing for 91.69% of members as of September, 2019. As the DHA adopts cross-service approaches to patient care, the ECHO model presents an ongoing opportunity to standardize care and share best practices. Not only is the ECHO model beneficial in leveraging specialty care access when geographic distance may prohibit a timely consultation, but it has proven to be beneficial during public health emergencies as well. When in-person patient and clinician safety are paramount, such as during the COVID-19 pandemic, the ECHO model has proven to be successful for specialty care consultation.^15^

In addition to the successes of ECHO telementoring in the military, the services have faced several challenges, which may need to be addressed if the model is to expand. The first is that initial champions for some subject areas may undergo changes of station at regular intervals, leaving gaps in hub team coverage or interest in maintaining the teleECHO sessions. The second is that videoconferencing software approved for military use is not as user-friendly as the Zoom platform, now used for the civilian ECHO experience. Thirdly, it is important that continuing education credits be optimized for all healthcare professionals as part of each ECHO program. This allows clinicians to both account for their time and to benefit from distance learning instead of needing to travel away from their post for an educational meeting. Finally, and probably most importantly, the decision to utilize the ECHO model requires the investment and commitment of military leadership if it is to endure among competing priorities for resources. Clinicians participating in the ECHO sessions, whether hub or spoke members, need protected time in order to continue to excel in their clinical activities and benefit from the educational venue of Project ECHO.

## CONCLUSIONS

Military ECHO participants at all levels model a concern for patient and peer well-being that is exemplary, and the pride they take in the care they deliver is well deserved. Each program has done a comprehensive job of making its content available across services, of collaborating in design and delivery of needed content, and of engaging a broad variety of clinical learners in acquiring new knowledge and skills. Military ECHO programs are integral parts of reducing the burdens of opioid overdose,^16^ diabetes, sexual assault, and amputation care among active and retired personnel and their dependents. These programs ensure that small and large MTFs can deliver a uniform standard of care while expanding the capacity of military primary and specialty clinicians to make effective use of limited resources.
